# Oversized nanodiscs for combined structural and functional investigation of multicomponent membrane protein systems

**DOI:** 10.1038/s41598-025-15035-3

**Published:** 2025-08-08

**Authors:** Bozhidar S. Ivanov, Judy Hirst

**Affiliations:** The Medical Research Council Mitochondrial Biology Unit, University of Cambridge, Keith Peters Building, Cambridge Biomedical Campus, Cambridge, UK

**Keywords:** Biochemistry, Biophysics, Biotechnology, Structural biology

## Abstract

Membrane proteins are fundamental to many crucial cellular processes but removing them from their native environment for structural and functional studies creates experimental challenges. Numerous strategies have been developed to replicate native-like membrane environments in vitro for membrane protein research, however, most studies have focused on systems for either structural or functional characterisation, not both together. Here, we apply an in-vivo split intein strategy to produce stable circularised nanodiscs for combined structural and functional analysis of respiratory complex I, using its highly hydrophobic native ubiquinone-10 substrate and an auxiliary ubiquinol oxidase from *Trypanosoma brucei brucei.* We successfully reconstituted *Paracoccus denitrificans* complex I into circularised nanodiscs, determined its cryo-EM structure at 3.1 Å resolution and conducted biophysical and biochemical analyses to demonstrate how the ‘oversized’ nanodiscs have space to accommodate both enzymes and substrates to sustain steady-state catalysis. Our work establishes a proof-of-principle for using oversized nanodiscs as an integrated platform for structural and functional interrogation of complex membrane proteins in near-native membrane environments.

**Supplementary Information:**

The online version contains supplementary material available at 10.1038/s41598-025-15035-3.

## Introduction

Membrane proteins (MPs) are critical components of the cell, performing important functions such as oxidative phosphorylation, transport of metabolites and ions, intra and inter cellular signalling, as well as membrane fusion and fission. They make up about 25% of human protein-encoding genes^1^ and have been estimated to represent over 60% of drug targets^2^. Despite their biological importance, the hydrophobic nature of MPs and challenges associated with their detergent-based isolation have limited the use of standard biophysical and biochemical techniques for their study, resulting in their structural and functional characterisation being under-represented compared to soluble proteins^3^. These challenges have also driven the development of membrane mimetic environments to isolate and stabilise MPs effectively. In recent decades, two primary strategies have gained prominence: one involving detergent-based isolation and reconstitution into membrane or membrane-like structures, such as amphipols^4^ membrane scaffolds (including nanodiscs^5–8^ and saposin A nanoparticles^9,10^ and proteoliposomes^11–13^; the other employing polymer-based technologies (such as styrene-maleic acid lipid particles [SMALPs]^14,15^, to isolate MPs but maintain their immediate native membrane environment. In particular, phospholipid bilayer nanodiscs (NDs) have emerged as promising and versatile membrane mimetics for the study of MPs^8,16^. They consist of phospholipids encircled by an amphipathic helical belt protein known as a membrane scaffold protein (MSP), constituting a small, stable phospholipid bilayer that offers enhanced MP stability by mimicking their natural environment^17^. Due to their ability to provide enhanced structural stability, NDs have become a widely used system for the structural study of MPs.

Many essential metabolic MPs depend on lipid-soluble substrates including ubiquinone-10, cholesterol, steroid hormones, fatty acids, cofactors, vitamins, and drugs. Recently, NDs have been used to study these MPs by co-incorporating their substrates into the ND bilayer. Important examples include respiratory CI^18–20^ and the alternative CIII^21^ various cytochrome P450 enzymes^22–24^ lipoxygenases^25^ and the STRA6 receptor^26^. While these studies have provided valuable insights into substrate binding and dynamics, the ND is typically too small to accommodate sufficient substrate for steady-state turnover, limiting their scope for studying catalysis. For example, NDs have been successful in stabilizing and resolving high-resolution cryo-EM structures of fragile and flexible bacterial CI complexes from *Escherichia coli*^20^ and *Paracoccus denitrificans*^19^ as well as the Q-bound structure of bovine CI^18^. However, these structures were limited to non-turnover conditions because the NDs could not accommodate enough ubiquinone-10 (Q_10_) or incorporate the auxiliary ubiquinol oxidase required to enable continuous turnover by recycling the product back into the substrate pool. For bovine CI in 2N2-NDs, the lack of catalytic activity could be further attributed to the MSPs wrapping too tightly around the membrane domain and restricting substrate exchange^18^ and the ~ 40% activity observed for *P. denitrificans* CI in 2N2-NDs was correlated to the proportion of larger detergent-ND bicelle hybrid particles observed by cryo-EM^19^. Together, these observations suggest that larger ND systems may offer new opportunities to comprehensively investigate enzyme mechanisms during catalysis.

While MSP-based NDs have been widely used, their diameter was, until recently, limited to around 16 nm^27^. The limitation arose from challenges in controlling the topology of how longer and less constrained MSPs form NDs with, for example, unequal MSP ‘wrapping’ to encircle unevenly sized discs, or the participation of different numbers of MPs. However, recent advances in ND engineering, such as the development of covalently circularised NDs (cNDs) and DNA-corralled NDs, have addressed these challenges and enabled the creation of homogeneous NDs with diameters of up to 50 and 70 nm, respectively^28,29^. Two key methods have emerged for producing cNDs: one involves an in-vitro approach in which MSPs are recombinantly expressed and circularised using sortase, yielding NDs up to 50 nm in diameter^28^ while the other is a simpler, high-yielding in-vivo split intein system that enables the production of cMSPs up to 26 nm diameter (cMSP26) in *E. coli*^30^. These advancements have opened new avenues for studying MPs, including investigating MP biogenesis^31–33^ receptor interactions^34–36^ viral entry^28,29,37^ and vaccine development^38,39^—but the potential of cNDs for studies of the catalytic functions of enzyme MPs during turnover has remained largely unexplored.

In this work, we leverage the in-vivo split intein method to produce cMSP26^30^ and develop a functional and robust cND system for investigating the catalysis of respiratory complex I using its native Q_10_ substrate and an auxiliary ubiquinol oxidase, AOX from *Trypanosoma brucei brucei*^40^. We successfully purified cMSP26 and reconstituted *P. denitrificans* complex I (*Pd*-CI) into the cMSP26 NDs forming *Pd*-CI-cNDs. We determined the *Pd*-CI cryo-EM structure within the cND at 3.1 Å resolution and conducted biophysical and biochemical analyses to demonstrate that the larger cND has space to accommodate both *Pd*-CI and AOX alongside each other. The resulting self-assembled cND respiratory units are a powerful tool for studying continuous catalysis of complex I by combining cryo-EM and functional strategies. Our results highlight the potential of the cND system to facilitate the co-reconstitution of multi-protein components for advanced studies of their structures and mechanisms.

## Results

### Preparation and characterisation of *Pd*-CI-cNDs

**Fig. 1:**
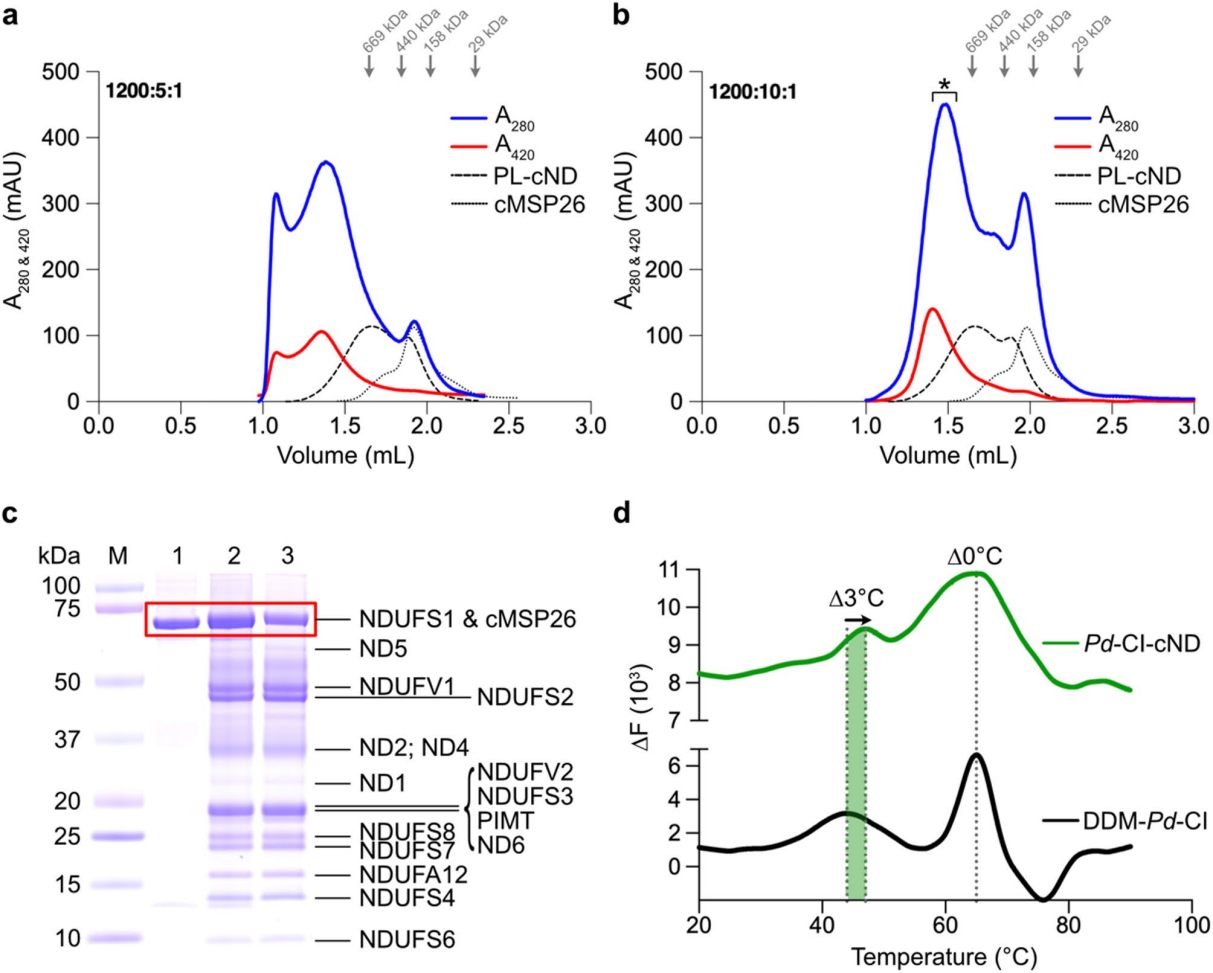
Biochemical and biophysical characterisation of *Pd*-CI-cNDs. Size exclusion chromatography (SEC) profiles for *Pd*-CI-cND reconstitutions: **a** *Pd*-CI-cND (1200:5:1, lipids: cMSP26:*Pd*-CI); and **b** *Pd*-CI-cND (1200:10:1, lipids: cMSP26:*Pd*-CI). Purified cMSP26 and PL-cNDs (*Pd*-CI-free cNDs) are included for comparison. Complex I absorbance due to the iron-sulphur clusters at 420 nm is shown in red. In **b**, the area under the peak used for cryo-EM grid preparation is indicated with a *. **c** SDS-PAGE analysis of the *Pd*-CI-cND sample used for cryo-EM. Lane 1: purified cMSP26; lane 2: *Pd*-CI-cND; and lane 3: *Pd*-CI purified in n-dodecyl-β-D-maltoside (DDM) as a reference. **d** Nano-DSF first derivative traces for DDM-purified *Pd*-CI and *Pd*-CI-cND.

The ability of our purified cMSP26 to circularise and enclose stable phospholipid bilayers (PL-cNDs) devoid of membrane proteins was first assessed using a molar ratio of 120 lipids:1 cMSP26. The size exclusion chromatography (SEC) traces following reconstitution of the bilayer showed a substantial increase in molecular weight (MW) relative to the purified cMSP26 alone (Fig. 1a). The main peak at 1.65 mL, corresponding to ~ 650 kDa, confirmed the formation of PL-cNDs. A smaller secondary peak at 2 mL coincided with the purified cMSP26 reference, indicating the presence of residual free cMSP26. Next, n-dodecyl-β-D-maltoside (DDM)-purified *Pd*-CI was reconstituted into cNDs using varying molar ratios of phospholipids, cMSP26, and *Pd*-CI. Initially, a molar ratio of 1200:5:1 (lipids to cMSP26 to *Pd*-CI) was tested. The SEC traces, confirmed by the complex I absorbance at 420 nm, shifted to higher molecular weight (1.45 mL elution volume) (Fig. 1a) compared to the detergent-solubilised *Pd*-CI standard (1.5 mL), indicating successful assembly of *Pd*-CI-cNDs. An additional peak at the column dead volume was also observed, likely due to soluble aggregates or higher-order molecular assemblies of around 5 MDa, possibly caused by excess phospholipids. Doubling the amount of cMSP26 to a molar ratio of 1200:10:1 eliminated the aggregation peak (Fig. 1b). Finally, SDS-PAGE analyses of the purified *Pd*-CI-cNDs sample verified the integrity of the *Pd*-CI within the cND (Fig. 1c).

The stability of the *Pd*-CI in the *Pd*-CI-cNDs was evaluated using nano-DSF (differential scanning fluorimetry) assays and compared to the detergent-purified complex. The first derivative traces for the *Pd*-CI reconstituted into cNDs were similar to those for *Pd*-CI in the smaller non-circularised MSP2N2-NDs (2N2-NDs) used previously^19^ showing two distinct peaks corresponding to the hydrophilic and membrane domains of *Pd*-CI (Fig. 1d). Notably, while the peak for the hydrophilic domain broadened but remained unchanged in position, the membrane domain of *Pd*-CI in cNDs displayed a 3 °C higher melting temperature than the detergent-purified enzyme, confirming the stabilising effect (Fig. 1d). Relative to 2N2-NDs^19^ the stabilization of the membrane domain in cNDs was slightly less pronounced, perhaps due to the larger ND size and greater degree of flexibility.

### Cryo-EM of *P. denitrificans* CI in cNDs

**Fig. 2:**
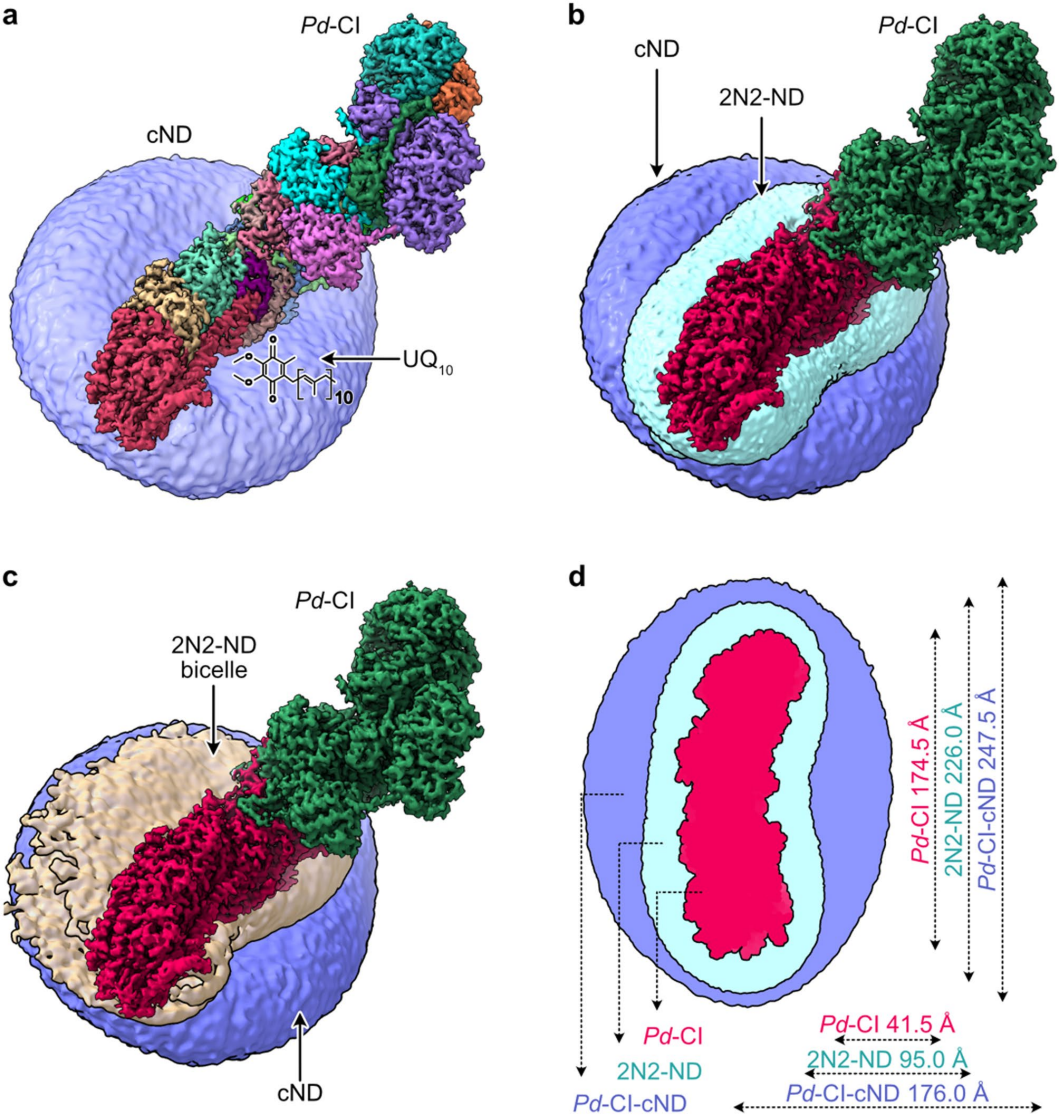
Interpretation of the cryo-EM density map for *Pd*-CI-cNDs. **a** The 3.1 Å-resolution cryo-EM density map of *Pd*-CI in cNDs coloured by subunit and presented within the Gaussian-smoothed large phospholipid cND bilayer (purple). **b** Comparison of the *Pd*-CI-cND map and the *Pd*-CI-2N2-ND map from Ivanov et al.^19^ used to determine the high-resolution structure of *Pd*-CI. The membrane-embedded portion of *Pd*-CI is coloured red and the hydrophilic domain green. **c** Comparison of *Pd*-CI in the cND with the hybrid DDM:2N2-ND bicelle observed in the 2N2-ND preparation. **d** Footprint comparison of *Pd*-CI to 2N2 and cND, with approximate maximum ‘diameters’ of length and width based on the map densities. Cryo-EM density maps were visualized using UCSF ChimeraX^57^ v1.6 [https://www.rbvi.ucsf.edu/chimerax/].

To prepare a sample for single-particle cryo-EM analysis, *Pd*-CI was reconstituted into cNDs containing Q_10_ (see Methods), purified by SEC to remove aggregates, CI-free cNDs, and free cMSP26 (Fig. 1b) and characterised as described above (Fig. 1c, d). The selected peak fractions (Fig. 1b) were combined and UltrAuFoil (0.6/1) grids prepared using undiluted samples at ~ 2 mg-protein mL^−1^. The grids were screened, and data were collected on a Titan Krios microscope operated in electron counting mode using a Gatan K3 detector and a post-column imaging energy filter and processed in *RELION 3.1.0*^41^ (Table S1 and Fig. S1). A final dataset of 57,305 particles led to a 3.1 Å resolution density map of *Pd*-CI reconstituted in cNDs (Fig. 2a).

Comparison of the cryo-EM densities for the 2N2-ND and cND systems revealed that the cNDs have substantially larger surface area and volume than the 2N2-NDs (Fig. 2b–d). The increase in size indicates that the larger lipid phase in cNDs accommodates more Q_10_ molecules, and that it may enable an auxiliary ubiquinol oxidase (AOX) to bind and form a fully functional minimal respiratory unit in which CI turns over continuously with its native Q_10_ substrate. Comparison of the measured area of the cryo-EM density for the *Pd*-CI-cNDs (Fig. 2d) with the area expected based on the 13 nm radius of the cMSP26 indicated that the density map does not capture the entirety of the disc area. Thus, using both values as limits, we subtracted the membrane footprint of *Pd*-CI (Fig. 2d) and estimated^40^ that each ND contains between 25 and 48 Q_10_ molecules. The equivalent calculation for *Pd*-CI-2N2-NDs is not possible as the MSP is linear and therefore may overlap itself^18^ (decreasing the size), or open^19^ (increasing the size). Previously, for our *Pd*-CI-2N2-NDs we observed a subpopulation of ‘open’ particles exhibiting an unusual detergent-ND bicelle structure (approximately twice the expected size, Fig. 2c). Importantly, while the MSP-enclosed 2N2-NDs lack space for binding an auxiliary ubiquinol oxidase, the expanded bicelle structure enabled interactions with AOX to facilitate turnover^19^. Similarly, in *Pd*-CI-cNDs the much larger area of the bilayer around the *Pd*-CI within the well-defined nanodisc assembly is expected to be sufficient to accommodate AOX for turnover.

### Continuous turnover of a minimal respiratory chain reconstituted into cNDs

**Fig. 3:**
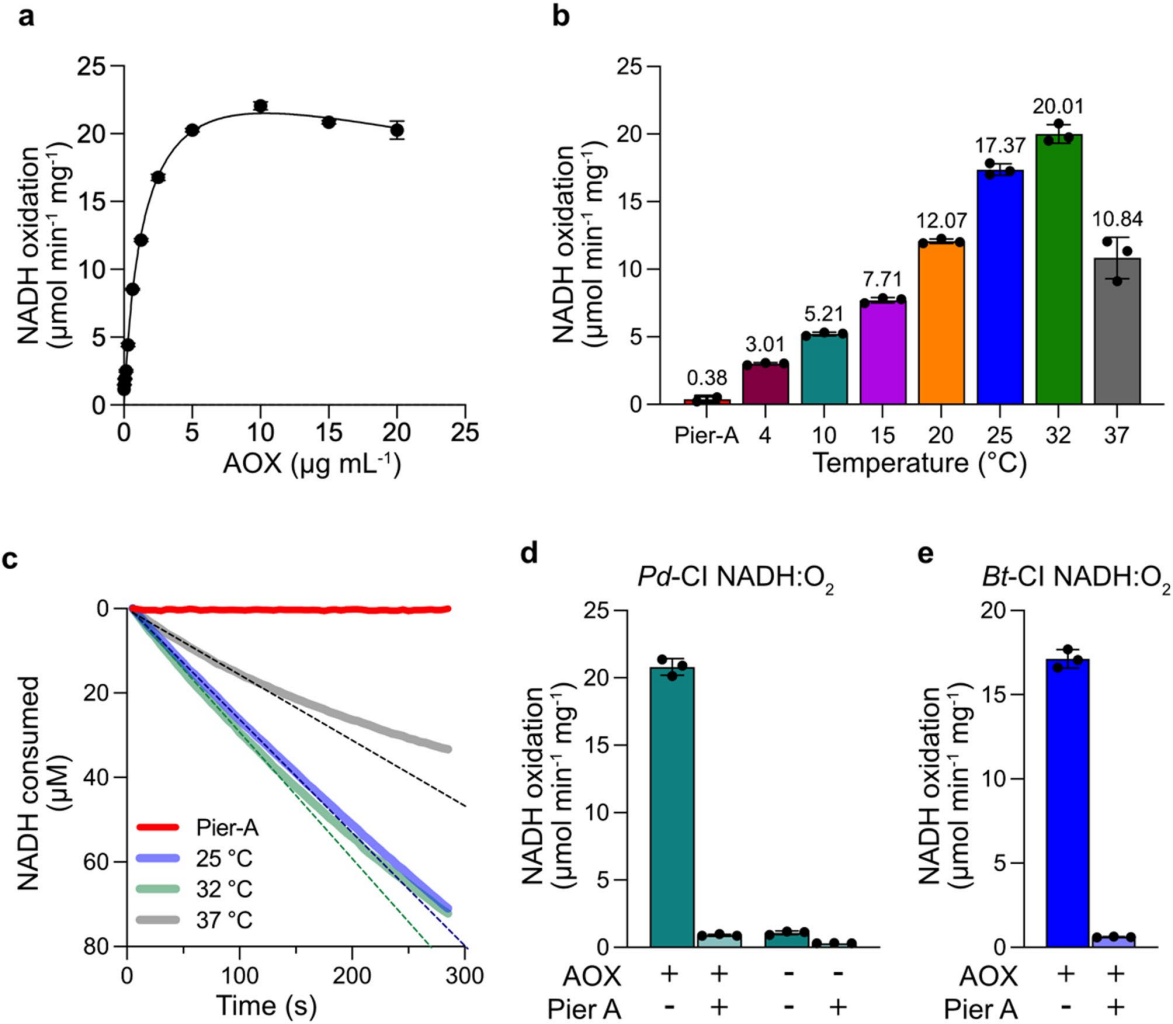
Continuous turnover in *Pd*-CI-cNDs. **a** NADH: O_2_ oxidoreduction by *Pd*-CI (0.5 µg mL^−1^) as a function of AOX concentration. **b** Temperature dependence of NADH: O_2_ oxidoreduction by *Pd*-CI-cNDs with AOX added at 10 µg mL^−1^. Initial rates were calculated by monitoring NADH oxidation during the first 2 min of the reaction. **c** NADH: O_2_ oxidoreduction traces for *Pd*-CI-cNDs with AOX at 25, 32, and 37 °C, with lines of best fit shown for the initial phase of each reaction. Pier-A controls at 4 °C are included in **b** and **c**. **d** and **e** show representative NADH: O_2_ oxidoreduction data from *Pd*-CI and bovine CI, respectively, reconstituted in cNDs and catalysing with 10 µg mL^−1^ AOX. See Methods for experimental details.

The catalytic activity of *Pd*-CI within cNDs was characterised as follows. First, the specific concentration of *Pd*-CI was determined using the NADH: APAD^+^ assay, which focussed on the NADH oxidation reaction in the hydrophilic domain. Then, the dependence of NADH: O_2_ oxidoreductase activity on the addition of AOX was assessed, with the optimal AOX concentration determined to be 10 µg mL^−1^ (Fig. 3a). This concentration (10 µg mL^−1^ AOX, or 20 µg AOX per µg CI) corresponds to the amount required when AOX was added to reconstituted *Pd*-CI-containing proteoliposomes under similar assay conditions^42^. Concentrations below 10 µg mL^−1^ AOX were rate limiting, whereas higher concentrations led to a gradual decrease in activity, potentially due to the increased amount of detergent, Mg ions and glycerol^5^ introduced by larger additions of AOX, which may interfere with either ND stability or CI activity (Fig. 3a).

Next, the temperature dependence of combined *Pd*-CI and AOX catalysis was evaluated from 4 to 37 °C. At lower temperatures (up to 20 °C) the NADH: O_2_ oxidoreduction activity was slower but sustained (Fig. 3b), and as the temperature increased the reaction rate also increased, with the maximum initial rate (20.0 ± 0.7 µmol NADH min^−1^ mg^−1^) achieved at 32 °C (Fig. 3b). Notably, the rates between 4 and 25 °C remained mostly constant throughout the 5-min reaction time, but at higher temperatures the reaction rate slows down over time with pronounced curvature observed at 37 °C after 100 s (Fig. 3c). The data suggest a balance between higher temperatures increasing catalysis kinetically but also compromising *Pd*-CI stability. Overall, the reconstituted combined system is able to catalyse across a wide temperature range, highlighting its potential for studying various MPs and their partners. For example, the lower rates observed at lower temperatures could be advantageous for studying fast enzyme kinetics, binding interactions, and other dynamic membrane processes.

The optimal catalytic rate for *Pd*-CI-cNDs, obtained at 32 °C using 200 µM NADH and 10 µg mL^−1^ AOX to recycle the Q_10_ present in the bilayer, was 20.8 ± 0.6 µmol NADH min^−1^ mg^−1^ from three independent reconstitutions (Fig. 3d), including the *Pd*-CI-cND cryo-EM sample (20.2 ± 0.1 µmol NADH min^−1^ mg^−1^). The observed NADH: Q_10_ rates were sensitive to piericidin A (a canonical complex I inhibitor), and no activity was detected in the absence of AOX (Fig. 3d). Concentrating the NDs had only a minor effect on catalysis (17.9 ± 0.1 µmol NADH min^−1^ mg^−1^ after concentration compared to 21.4 ± 0.2 beforehand), which is a helpful indicator for the handling of low abundance samples. Finally, to further stretch the system, we extended it to the larger and more complex mitochondrial CI by reconstituting bovine CI (purified in lauryl maltose neopentyl glycol-LMNG) into cNDs. The observed piericidin A-sensitive rate of catalysis of 17.1 ± 0.6 µmol min^−1^ mg^−1^ (Fig. 3e) is consistent with values reported for the established CI proteoliposome system, reflecting comparable catalytic functionality^13,40,43^. The larger footprint of the mammalian enzyme within the membrane did not hinder substrate availability or the interaction of AOX with the cND. Our results establish the cND system as a universally applicable platform for complex I from both prokaryotic and eukaryotic species.

### Modelling AOX binding to *Pd*-CI-cNDs

**Fig. 4:**
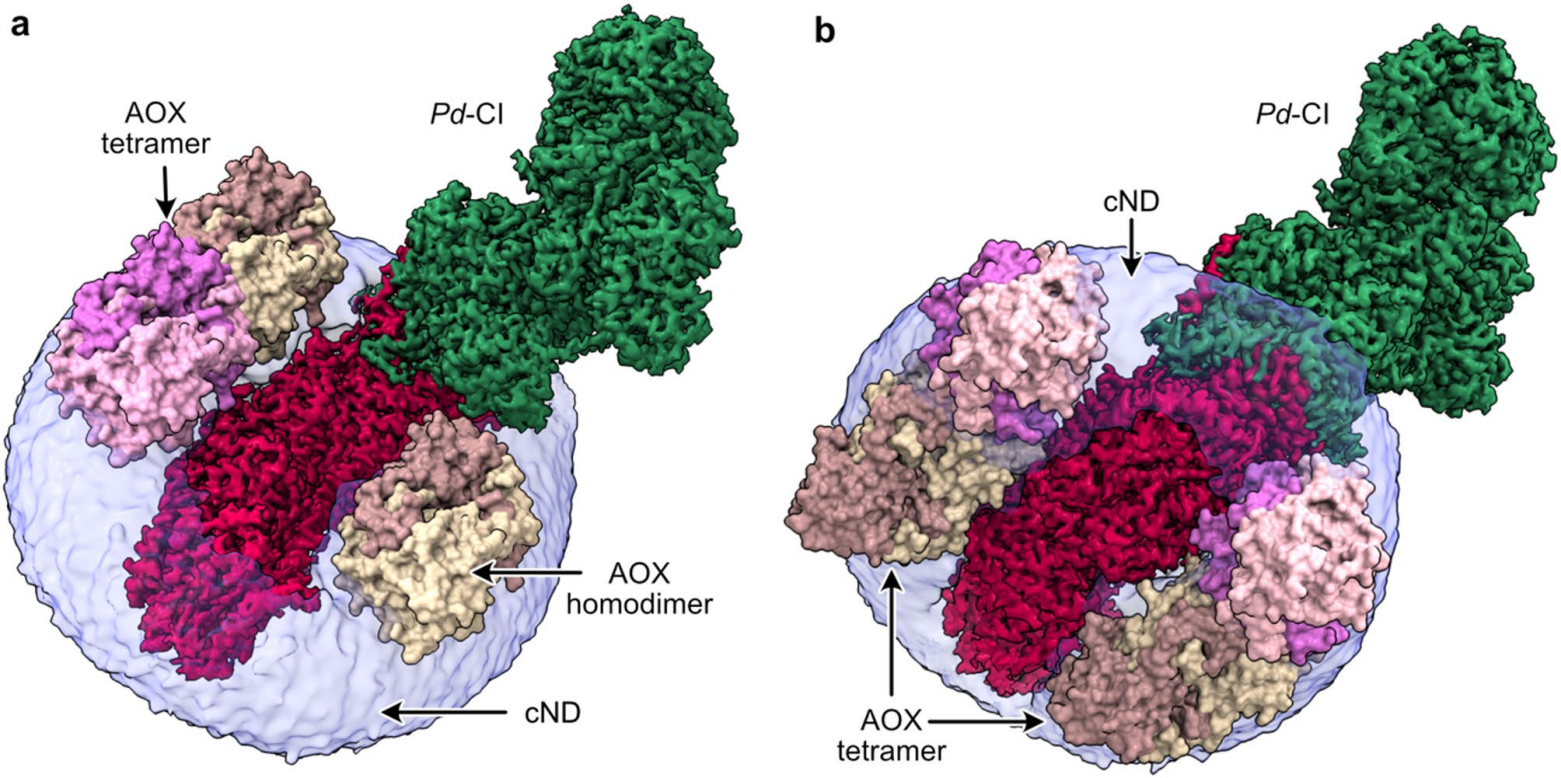
Proposed structure-function relationships of *Pd*-CI and AOX in the continuous turnover cND system. Predicted **a** top and **b** bottom views of dimeric and tetrameric AOX peripherally bound to either leaflet of the *Pd*-CI-cND. The membrane and hydrophilic domains of the *Pd*-CI density map are coloured red and green, respectively, and displayed within the Gaussian-smoothed cryo-EM density for the cND bilayer, shown in semi-transparent purple. The crystal structure of the *T. brucei* AOX (PDB-3W54^44^) is shown as solid surface that has been computationally docked to either leaflet of the phospholipid bilayer. The AOX is shown binding in dimeric and tetrameric assemblies, with the monomers shown in different colours. Cryo-EM density maps were visualized using UCSF ChimeraX^57^ v1.6 [https://www.rbvi.ucsf.edu/chimerax/].

The structure of the AOX from *T. brucei brucei* (PDB-3W54^44^) was docked to the lipid bilayer of the *Pd*-CI-cNDs (using the cryo-EM density) to visualise the accessibility of AOX binding. Structural data suggest that AOX is a functional dimer and possibly even a tetramer^44^ thus both structures were considered (Fig. 4). The analysis shows that there is enough physical space for a homodimer and tetramer to bind on the top cND phospholipid leaflet (Fig. 4a) and either four dimers or two tetramers on the bottom leaflet (Fig. 4b). Thus, AOX may bind to the phospholipid bilayer to allow continuous CI turnover, demonstrating the opportunity to create multi-component systems and extending the experimental scope for structure-function studies of MPs in near native environments.

## Discussion

The modular *Pd*-CI-cNDs system described here combines the advantages of a purified enzyme with the ability to catalyse with a hydrophobic substrate in a near-native membrane environment for combined biochemical, biophysical and structural studies. *Pd*-CI-cNDs enable formation of a minimal respiratory chain designed to study continuous complex I catalysis with its native lipid-soluble Q_10_ substrate incorporated in the membrane, and gain added stability towards changes in pH^45^ ionic strength^46^ temperature^30^ and membrane rigidity^47^ from the MSP circularisation. Here, we reconstituted *Pd*-CI into circularised NDs, resolved its cryo-EM structure in the disc, and augmented the composition with AOX to assemble a minimal respiratory unit capable of sustained catalysis. Although the fundamental properties of *Pd*-CI in cNDs closely resemble those described by Ivanov et al.^19^ for *Pd*-CI reconstituted in 2N2-NDs (high thermal stability, matching CI structure evidenced by > 95% map correlation) the ‘oversized’ nature of the cNDs offers the new opportunity to incorporate the auxiliary enzyme, plus the advantage of a single, homogeneous ND population. We further expanded our study to reconstitute the larger mammalian CI into cNDs and enable steady-state catalysis using the same approach: the observed rates of NADH: O_2_ oxidoreduction were comparable to those achieved in proteoliposome systems^48^. In contrast, reconstitution of bovine CI into 2N2-NDs wrapped the enzyme too tightly and blocked substrate exchange^18^ while reconstitution of *Pd*-CI into 2N2-NDs yielded an additional population of larger ND-bicelle hybrid particles^19^ suggesting the partial opening of the linear MSP enclosure. The precise control over size, structure and phospholipid area possible for cNDs thus highlights the opportunity of using them to create multiprotein membrane assemblies tailored to specific research needs. Specifically, our co-reconstituted, homogeneous and well-defined oversized cNDs now provide an optimal platform for capturing structures of complex I under turnover^19^. More broadly, cNDs offer sufficient membrane space to study stepwise assembly and biogenesis of large complexes, as shown for the SNARE complex^49^ and are well-suited for investigating protein-protein, protein-lipid, and protein-drug interactions, including viral entry studies^28^. Recent studies further highlight their potential as platforms for MP-targeted antibody production and drug delivery, owing to their enhanced stability in-vivo^50^.

In summary, respiratory complex I provides a proof-of-principle demonstration for the use of oversized cNDs to study membrane protein structure and function in a near-native environment, suggesting their potential application in studies of a broad range of membrane proteins, in the presence of highly hydrophobic substrates, drugs, and therapeutic compounds. Their unique combination of stability, high substrate capacity, and compatibility with complex membrane protein systems underscores their potential to advance membrane protein biology.

## Materials and methods

All chemicals and reagents were purchased from Merck unless stated otherwise.

### Preparation of complex I from *P. denitrificans*

*P. denitrificans* membranes were prepared according to the protocol described by Ivanov et al.^19^. 12 × 2 L Erlenmeyer flasks containing 500 mL of fresh LB broth were inoculated with 0.1% (v/v) of overnight pre-culture of the *Pd-*Nqo5^His6^ strain^51^. Cells were grown aerobically at 30 °C with 225 rpm shaking and harvested at late-log phase (OD_600_ 4 to 4.5) by centrifugation (5000*×g*, 30 min, 4 °C). All subsequent steps were performed at 4 °C. Cells were resuspended and homogenised in ~ 25 mL of lysis buffer (50 mM MES, pH 6.5 at 4 °C, plus one cOmplete™ EDTA-free protease inhibitor cocktail tablet (Roche) per 50 mL) per gram of wet cell pellet and lysed using a Z-plus 2.2 kW cell disruptor (Constant Systems Limited), once at 15,000 psi and twice at 25,000 psi. Cell debris were removed by centrifugation (31,900*×g*, 1 h) and membranes harvested by ultracentrifugation (234,800×*g*, 2 h), resuspended in 50 mM MES buffer (pH 6.5 at 4 °C), flash frozen in liquid nitrogen (LN_2_) and stored at – 70 °C.

Complex I was prepared using the protocol described by Ivanov et al.^19^ with minor modifications. Membranes (~ 250 mg protein) were thawed and adjusted to 10 mg mL^−1^ in buffer containing 20 mM MES (pH 6.5 at 4 °C), 200 mM NaCl, 2 mM CaCl_2_, 20 mM imidazole, 10% (v/v) glycerol, and the cOmplete™ EDTA-free protease inhibitor cocktail. Solubilisation was achieved by dropwise addition of *n*-dodecyl-β-D-maltoside (DDM, Anatrace) to a final concentration of 3%, and stirred at 4 °C for 30 min. Insoluble material was removed by centrifugation (172,000×*g*, 45 min), then the supernatant was passed through a 0.45 μm syringe filter (Millex^HP^, Millipore) and loaded onto a 5 mL His-Trap™ HP column (Cytiva) equilibrated with buffer A (20 mM MES (pH 6.5 at 4 °C), 100 mM NaCl, 2 mM CaCl_2_, 20 mM imidazole, 10% (v/v) glycerol, 0.1% (w/v) DDM, 0.02%(w/v) soy bean asolectin (Avanti Polar Lipids) and 0.02% (w/v) 3-[(3-cholamidopropyl)dimethylammonio]-1-propane-sulfonate (CHAPS). The column was washed with a gradient of 20–40 mM imidazole over seven column volumes, before eluting CI with elution buffer B (buffer A containing 300 mM imidazole). Complex I-containing fractions were pooled, concentrated to 1 mL (100 kDa MW cut-off, Merck Millipore), filtered, and loaded onto a Superdex 200 Increase 10/300 GL size exclusion column (Cytiva), equilibrated with buffer C (20 mM MES (pH 6.5 at 4 °C), 100 mM NaCl, 2 mM CaCl_2_, 10% (v/v) glycerol, 0.05% (w/v) DDM). Complex I-containing fractions were pooled and concentrated to 15–20 mg mL^− 1^ before glycerol was added to a final concentration of 30% (v/v) and the protein was flash frozen in LN_2_ and stored at -70 °C.

### Preparation of the alternative oxidase (AOX)

Recombinantly expressed AOX was prepared from *E. coli* membranes by solubilisation with octyl-glucoside and purified by Twin-Strep tag affinity chromatography as described previously^52^. Membranes were solubilised at 6 mg mL^−1^ in buffer containing 25 mM Tris-HCl pH 7.5 (4 °C), 200 mM MgSO_4_ and 20% (v/v) glycerol by dropwise addition of 1.4% (w/v) octyl-glucoside (OG) for 1 h at 4 °C. All subsequent steps were performed at 4 °C. The solubilised material was collected by centrifugation (230,000×*g*, 30 min), syringe filtered (0.45 μm filter Millex^HP^ Millipore) and loaded onto a 10 mL Strep-Tactin^®^ Superflow^®^ high-capacity column. The column was washed with AOX buffer A (20 mM Tris-HCl pH 7.5 (4 °C), 50 mM MgSO_4_, 160 mM NaCl, 0.042% (w/v) DDM and 20% (v/v) glycerol) before eluting the protein with AOX buffer B (buffer A supplemented with 2.5 mM desthiobiotin). AOX was concentrated (10 kDa MWCO filter, Amicon ^®^ Ultra, Merck) and dialysed into buffer A before it was flash frozen in LN_2_ and stored at – 70 °C.

### Preparation of cMSP26

cMSP26 was prepared according to a protocol adapted from Miehling et al.^30^. Erlenmeyer flasks containing 500 mL of fresh LB broth supplemented with 50 µg mL^−1^ kanamycin and 25 µg mL^−1^ chloramphenicol were inoculated with 0.1% (v/v) pre-culture of *E. coli* BL21-CodonPlus RIL (DE3) containing cMSP26 plasmid and grown aerobically (37 °C, 225 rpm) until OD_600_ of 0.6. Protein expression was induced by 0.1 mM isopropyl β-d-1-thiogalactopyranoside (IPTG) and the cells were cultured for further 16–20 h at 25 °C with shaking. Cells were harvested by centrifugation (6000×*g*, 20 min), resuspended in lysis buffer (50 mM Tris-HCl pH 8, 400 mM NaCl, 10 mM imidazole, 5 mM MgCl_2_, cOmplete™ EDTA-free protease inhibitor cocktail, and a few crystals of DNase I) and lysed by passing the suspension three times through a French press at 20,000 psi or by sonication using a Qsonica sonicator (60% power, 20-second cycles on and off, 20 min total sonication time). All subsequent steps were performed at 4 °C. The lysate was clarified by ultracentrifugation (200,000*×g*, 1 h) syringe filtered (0.22 μm, Millex^HP^ Millipore) and applied to a 5 mL HisTrap™ HP column (Cytiva) equilibrated with MSP buffer A (20 mM Tris-HCl pH 8.0, 200 mM NaCl, 10 mM imidazole). The column was washed with MSP buffer A until the A_280_ absorbance reached baseline, followed by sequential washes with five column volumes of MSP buffer A containing 20 mM imidazole and five volumes containing 40 mM imidazole. The protein was eluted with MSP buffer A containing 500 mM imidazole, peak fractions were pooled and concentrated (100 kDa MWCO filter, Amicon ^®^ Ultra, Merck) before exchanging the buffer for storage (20 mM Tris pH 7.4, 200 NaCl) using dialysis or a desalting PD10 column (Cytiva). Protein was flash frozen in LN_2_ and stored at – 70 °C.

### Reconstitution of *Pd*-CI into circularised phospholipid nanodiscs (*Pd*-CI-cNDs)

cNDs were prepared using a protocol adapted from Ivanov at al^19^. Lipids (0.5 mg; 8:1:1 mixture of 1,2-dioleoyl-*sn*-glycero-3-phosphocholine (PC), 1,2-dioleoyl-sn-glycero-3-phosphoethanolamine (PE), and 18:1 cardiolipin (CDL), Avanti Polar Lipids) in chloroform were combined with Q_10_ (50 nmol per mg lipid), then placed under a N_2_ stream to remove the solvent and dried under vacuum for 2 h. The lipids/Q_10_ mixture was hydrated in ND reconstitution buffer (0.5 mL; 20 mM MES pH 6.5, 25 mM NaCl) at 1 mg mL^−1^ for 30 min with frequent vortexing. Sodium cholate was added to 40 mM, and the mixture sonicated in a bath sonicator for 10 min. All subsequent steps were performed at 4 °C. cMSP26 and *Pd*-CI were added to the lipid/Q_10_ mixture at a molar ratio of 1200 lipids:10 cMSP26:1 *Pd*-CI, diluted to 0.5 mg mL^−1^ lipids and incubated on ice for 20 min. Sodium cholate was removed using a PD10 desalting column, and the NDs were concentrated to 100 µL (100 kDa MW cut-off concentrator, Merck Millipore) and purified using Superose 6 Increase 5/150 size exclusion column on an AKTA Micro system (Cytiva). The total protein concentration (*Pd*-CI and cMSP26) was determined using the Pierce™ bicinchoninic acid (BCA) assay (Thermo Fisher Scientific).

### Kinetic measurements

All kinetic assays were carried out at 32 °C in a Molecular Devices SpectraMax ABS Plus 96-well plate reader in buffer containing 10 mM MES or MMT (MES, MOPS, Tris) pH 6.5 at 32 °C, 25 mM NaCl and 2 mM CaCl_2_. The *Pd*-CI content in *Pd*-CI-NDs was quantified by comparison to a standard sample of *Pd*-CI in DDM using the NADH: APAD^+^ oxidoreduction assay (where APAD^+^ is 3-acetylpyridine adenine dinucleotide) with 100 µM NADH, 500 µM APAD^+^, 1 µM piericidin A and 0.1% DDM, monitored at 400 and 450 nm (ε_(400–450 nm)_ = 3.16 mM^−1^ cm^−1^). NADH: O_2_ oxidoreduction by *P. denitrificans* membranes was measured using 200 µM NADH and 10 µg mL^−1^ alamethicin following NADH absorbance at 340 and 380 nm (ε_(340–380 nm)_ = 4.81 mM^−1^ cm^−1^). NADH: O_2_ oxidoreduction by *Pd*-CI-NDs was measured using 200 µM NADH and 10 µg mL^−1^ AOX^40,53^. NADH: decylubiquinone (dQ) oxidoreductase activity was typically measured in 200 µM NADH, 200 µM dQ, 0.15% soybean asolectin and 0.15% CHAPS.

### Nano-DSF

*Pd*-CI-DDM and *Pd*-CI-ND were diluted to 0.2 mg mL^−1^ in buffer C and ND reconstitution buffer, respectively, loaded into capillaries and placed in a Prometheus NT.48 instrument (NanoTemper Technologies). The temperature was raised from 20 to 90 °C at 4.5 °C min^−1^ and the tryptophan fluorescence monitored at 330 and 350 nm using an excitation wavelength of 280 nm^54^.

### Cryo-EM grid Preparation and data acquisition

UltrAuFoil 0.6/1 grids (Quantifoil Micro Tools GmbH, Germany) were prepared as described previously^55^. Grids were glow discharged (20 mA for 90 s), incubated under anaerobic conditions in 5 mM 11-mercaptoundecyl hexaethylene glycol (TH 001-m11.n6-0.01, ProChimia Surfaces) in ethanol for 48 h, then washed several times with 100% ethanol and air dried. 2.5 µL of 2.25 mg mL^−1^ *Pd*-CI-NDs were applied to each grid in an FEI Vitrobot IV (Thermo Fisher Scientific) at 4 °C and 100% relative humidity before blotting for 10 s with a blot force setting of − 10 and vitrifying the sample in liquid ethane. Data were screened and acquired using a Gatan K3 detector and energy filter (Gatan BioContinuum) operated in zero-loss mode with a slit width of 20 eV on a 300 keV FEI Titan Krios microscope (Thermo Fisher Scientific) at the cryo-EM facility at the Department of Biochemistry, University of Cambridge. Imaging was performed with a 100 μm objective aperture and a 70 μm C2 aperture and data were recorded using EPU software. *Pd*-CI-ND images were collected in counting mode at a nominal magnification of 64,000*×*, giving a facility-provided pixel size of 1.43 Å pixel^−1^ (later calibrated to 1.34 Å pixel^− 1^) with defocus ranges of − 0.9 to − 2.5 μm in 0.1 μm increments and the autofocus routine run every 10 μm. The dose rate was 12.29 *e*^*−*^ Å^−2^ s^−1^ with 3.4 s exposure giving total doses of 41.8 *e*^*−*^ Å^−2^ captured in 40 frames. All data were acquired as one shot per hole with 5 s delay after stage shift and 1 s delay after image shift.

### Cryo-EM image processing

The *Pd*-CI-ND dataset was processed using RELION 3.1.0^41^. RELION’s implementation of motion correction was used with 2 x binning and 5 × 5 patches, and the CTF estimated using CTFFIND-4.1^56^ with an amplitude contrast of 0.1 and maximum resolution of 4 Å. Following filtering to remove micrographs with CtfFigureOfMerit <− 0.05, MaxResolution > 7.5 Å, and CtfAstigmatism < 25 or > 1000, ice-contaminated micrographs were removed manually. From the resulting 1101 micrographs, 450,598 particles were picked using *RELION’s AutoPicking* tool and 2D map references of bovine complex I. The particles were extracted with a box size of 450 pixels, downscaled to 200 pixels, and subjected to initial 3D global classifications to remove junk particles. The strong density of the large NDs was subtracted and the particles were re-extracted at nominal pixel size of 1.43 Å pix^−1^ and further classified by 3D global classifications with searches to 3.7° yielding 78,992 good CI particles. Then, the particles were 3D refined, followed by iterative 3D refinements and CTF refinements (for anisotropic magnification, beam-tilt, trefoil, and per particle defocus, astigmatism and B-factor correlation) and Bayesian polishing (per-particle motion correction) at 1.43 Å pix^−1^ before a final 3D classification with local angular searches at 3.7, 1.8 and 0.9° was carried out to obtain 76,926 particles. Poor density was noted for the distal section of the membrane domain and focussed 3D classifications without angular searches were performed using a low-pass filtered 15 Å-resolution mask with a soft edge of 10 pixels, which was created from a map generated using the *molmap* function in UCSF ChimeraX^57^ using ND5 subunit from 8YBY model^19^. The resulting 57,305 particles were 3D refined and post-processed at a calibrated pixel size of 1.34 Å pix^− 1^ to produce a final map with a global resolution of 3.1 Å.

## Supplementary Information

Below is the link to the electronic supplementary material.

Supplementary Material 1

Supplementary Material 2

## Data Availability

Structural data have been deposited in the EMDB database under the accession code EMD-53583 [https://www.ebi.ac.uk/emdb/EMD-53583]. The cryo-EM raw images are available from EMPIAR with the accession code EMPIAR-12775 [https://www.ebi.ac.uk/empiar/EMPIAR-12775/]. Data supporting the findings of this study are available from the corresponding author upon reasonable request. Please contact Professor Judy Hirst at jh480@cam.ac.uk for data access inquiries.
