# Supplementary figures and images for: Oversized nanodiscs for combined structural and functional investigation of multicomponent membrane protein systems
Source: Sci Rep. 2025 Aug 8;15:29070. doi: 10.1038/s41598-025-15035-3 (PMC12334642; doi:10.1038/s41598-025-15035-3)

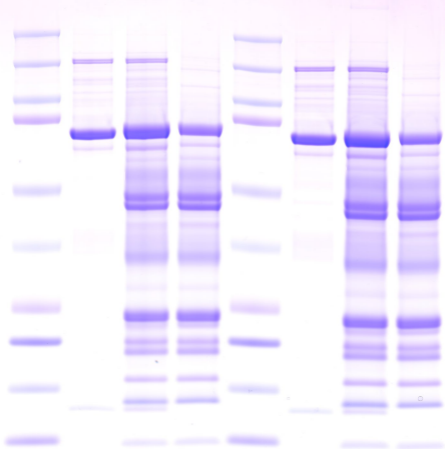

Supplement: Supplementary file 1 — Supplementary Material 1 [file 41598_2025_15035_MOESM1_ESM.pdf]
