# Supplementary material for: Oversized nanodiscs for combined structural and functional investigation of multicomponent membrane protein systems
Source: Sci Rep. 2025 Aug 8;15:29070. doi: 10.1038/s41598-025-15035-3 (PMC12334642; doi:10.1038/s41598-025-15035-3)
Supplement: Supplementary file 2 — Supplementary Material 2 [file 41598_2025_15035_MOESM2_ESM.docx]

Supplementary Information

**Oversized nanodiscs for combined structural and functional investigation of multicomponent membrane protein systems**

Bozhidar S. Ivanov and Judy Hirst*

The Medical Research Council Mitochondrial Biology Unit, University of Cambridge, Keith Peters Building, Cambridge Biomedical Campus, Cambridge, United Kingdom

*e-mail: [jh480@cam.ac.uk](mailto:jh480@cam.ac.uk)

**This file includes:**

Supplementary Figure 1

Supplementary Table 1

**Figure S1**

**
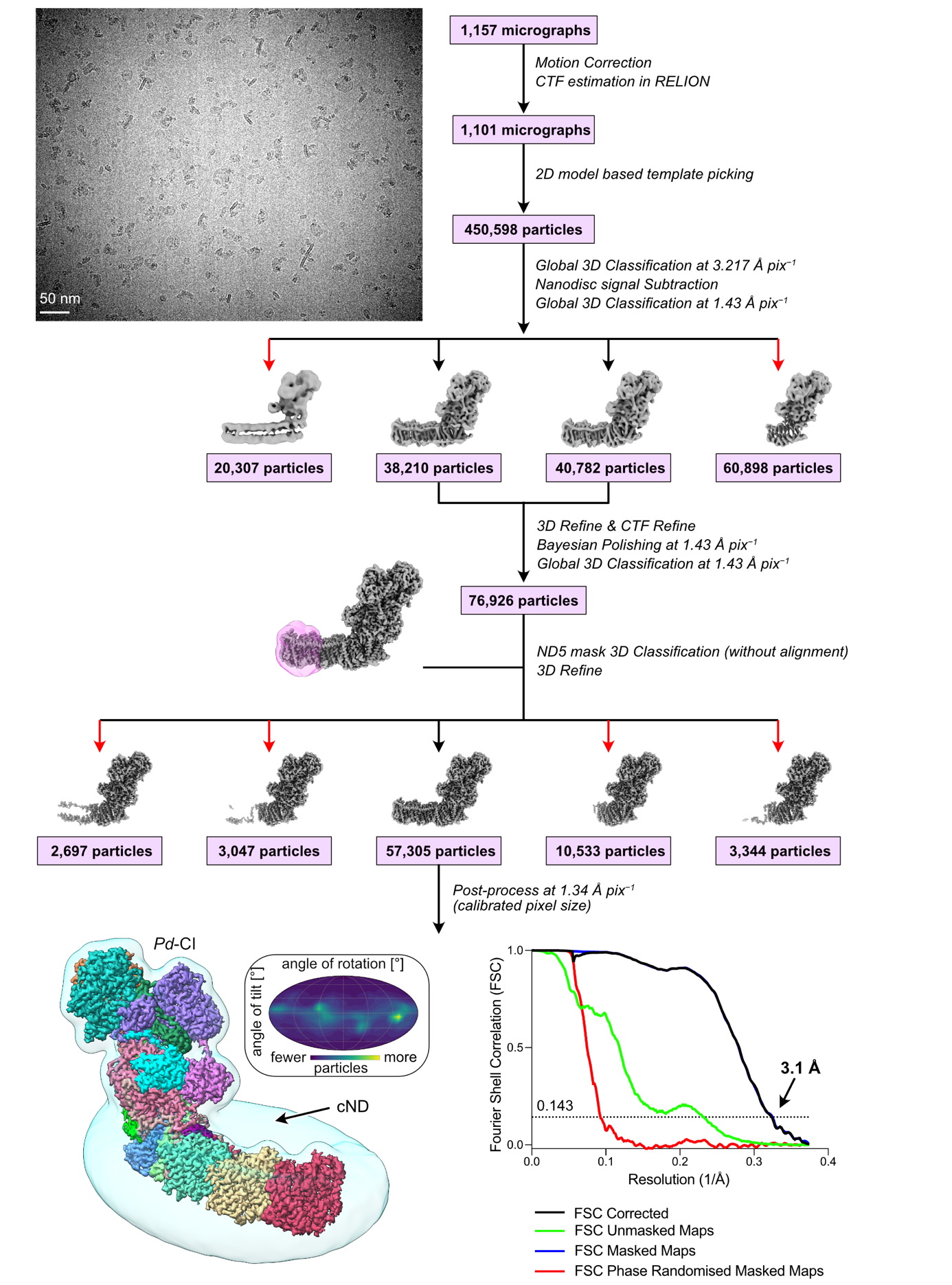
**

**Figure S1 | Cryo-EM data processing scheme for *Pd*-CI-cNDs.** A flowchart showing the pipeline employed to obtain the final map for *Pd*-CI-cNDs. An example micrograph and 3D classes, along with the ND4 and ND5 mask (semi-transparent pink) are shown. Red arrows indicate particles that were excluded. The final map is colour-coded by subunit and shown along with the Mollweide plot for orientation distributions and Fourier Shell Correlation (FSC) curves. Cryo-EM data were collected using EPU software (Thermo Fisher Scientific, v2.10.0.5). Cryo-EM density maps were visualized using UCSF ChimeraX^57^ v1.6 [<https://www.rbvi.ucsf.edu/chimerax/>].

**Table S1 | Cryo-EM data collection and refinement statistics.**

| **Data collection and processing** | ***Pd*-CI-cNDs** |
| --- | --- |
| Voltage (kV) | 300 |
| Nominal magnification | 64,000× |
| Electron exposure (*e^–^* Å^-2^) | 41.80 |
| Defocus range (μm) | -0.9 to -2.3 |
| Calibrated pixel size (Å) | 1.34 |
| Number of frames | 40 |
| Symmetry imposed | C1 |
| Number of micrographs | 1,151 |
| Initial particle images (no.) | 450,598 |
| Final particle number (no.) | 57,305 |
| Map sharpening *B* factor (Å^2^) | -38.56 |
| Map resolution (Å) (FSC = 0.143) | 3.1 |
| Map resolution range (Å) | 2.8 – 7.2 |
